# Complete Genome Sequences of the Plant Pathogens *Dickeya solani* RNS 08.23.3.1.A and *Dickeya dianthicola* RNS04.9

**DOI:** 10.1128/genomeA.01447-17

**Published:** 2018-01-25

**Authors:** Slimane Khayi, Pauline Blin, Teik Min Chong, Kévin Robic, Kok-Gan Chan, Denis Faure

**Affiliations:** a Institute for Integrative Biology of the Cell (I2BC), CEA CNRS Université Paris-Sud, Université Paris-Saclay, Gif-sur-Yvette, France; b Biotechnology Research Unit, National Institute for Agronomic Research (INRA), Rabat, Morocco; c Division of Genetics and Molecular Biology, Institute of Biological Sciences, Faculty of Science, University of Malaya, Kuala Lumpur, Malaysia; d Semences, Innovation, Protection, Recherche, Environnement (SIPRE) Achicourt, France

## Abstract

*Dickeya* spp. are bacterial pathogens causing soft-rot and blackleg diseases on a wide range of ornamental plants and crops. In this paper, we announce the PacBio complete genome sequences of the plant pathogens *Dickeya solani* RNS 08.23.3.1.A (PRI3337) and *Dickeya dianthicola* RNS04.9.

## GENOME ANNOUNCEMENT

Pectinolytic enterobacteria belonging to the genera *Dickeya* and *Pectobacterium* are worldwide pathogens that are responsible for blackleg and soft-rot diseases on several crops and ornamental plants (1–3). *Dickeya dianthicola*, which was initially reported on ornamental plants of the genus *Dianthus*, was identified after observation of infection symptoms on *Solanum tuberosum* in the 1970s. More recently, *Dickeya solani* emerged in the 2000s as a novel potato pathogen in several European countries (4).

Draft genome sequences of *Dickeya solani* RNS 08.23.3.1.A and *Dickeya dianthicola* RNS04.9 were previously generated from Illumina HiSeq 2000 version 3 sequencing of 8-kbp mate-pair libraries (5, 6). To fix the assembly issues that are commonly associated with short reads, their genomes were sequenced using the PacBio sequencing RS II platform (Pacific Biosciences, CA, USA). DNA extractions were performed from overnight cultures using a MasterPure DNA purification kit (Epicentre, USA). Quantification and quality control of the DNA were completed using a NanoDrop ND1000 device, Qubit 2.0 fluorometer, and 1.0% agarose gel electrophoresis gels. The library had 10-kbp insert sizes. Prior to assembly, short reads that are less than 500 bp were filtered off, and the minimum polymerase read quality used for mapping of subreads from a single zero-mode waveguides was set at 0.75. A total of 112,228 filtered reads for *D. solani* RNS 08.23.3.1.A (*N*_50_, 13,159 bp) and 122,395 filtered reads (*N*_50_, 10,699 bp) for *D. dianthicola* RNS04.9 were obtained. The filtered reads were assembled using RS_HGAP_Assembly software version 2.0. The cutoff lengths of seeding reads were set at 3,606 bp for *D. solani* and 4,247 bp for *D. dianthicola* RNS04.9 in order to serve as a reference for the recruitment of shorter reads for preassembly. The resulting consensus accuracy based on multiple-sequence alignment of the subreads was at 99.99%. By combining the sequences of Illumina sequencing and PacBio sequencing, the chromosomal anatomy of these strains was completely resolved. The genome sequences for both strains were annotated using the Rapid Annotations using Subsystems Technology (RAST) version 4.0 automated pipeline (7). The *D. solani* circular genome is 4,922,460 bp (56.3% GC content) and contains 4,536 coding sequences (CDSs), 75 tRNAs, and 22 rRNAs, while the *D. dianthicola* circular genome size is 4,720,132 bp (56.0% GC content), with 4,567 CDSs, 74 tRNAs, and 22 rRNAs.

### Accession number(s).

The complete genome sequence projects for these bacteria have been deposited at DDBJ/EMBL/GenBank under the accession numbers CP016928 (*Dickeya solani* RNS 08.23.3.1.A) and CP017638 (*Dickeya dianthicola* RNS04.9).
